# Triglyceride–glucose index change and chronic kidney disease progression in a Chinese hypertensive population

**DOI:** 10.3389/fendo.2024.1342408

**Published:** 2024-02-09

**Authors:** Chao Yu, Yumeng Shi, Tao Wang, Lingjuan Zhu, Wei Zhou, Huihui Bao, Xiaoshu Cheng

**Affiliations:** ^1^ Department of Cardiovascular Medicine, The Second Affiliated Hospital, Jiangxi Medical College, Nanchang University, Nanchang, Jiangxi, China; ^2^ Center for Prevention and Treatment of Cardiovascular Diseases, The Second Affiliated Hospital, Jiangxi Medical College, Nanchang University, Nanchang, Jiangxi, China; ^3^ Jiangxi Provincial Cardiovascular Disease Clinical Medical Research Center, Nanchang, Jiangxi, China; ^4^ Jiangxi Sub-Center of National Clinical Research Center for Cardiovascular Diseases, Nanchang, Jiangxi, China

**Keywords:** triglyceride glucose index change, chronic kidney disease progression, hypertension, prospective cohort study, estimated glomerular filtration rate

## Abstract

**Background:**

The impact of triglyceride–glucose (TyG) index variations on chronic kidney disease (CKD) progression remains unexplored. To investigate the effects of the TyG index and its dynamic changes on CKD progression.

**Method:**

This prospective cohort study included data from 8,418 hypertensive participants. The exposure variable in this study was defined as the difference between the TyG index at the last visit from that at baseline. The study’s outcome variable was the progression of CKD, defined as follows: for subjects with an estimated glomerular filtration rate (eGFR) ≥60 mL/min, a ≥30% decrease in eGFR with a final follow-up value <60 mL/min; for those with an eGFR <60 mL/min, a ≥50% decrease in eGFR; or terminal renal failure requiring dialysis.

**Results:**

During a median follow-up period of 48 months, 1077 patients were diagnosed with CKD progression. In the fully adjusted Model 3, patients with a change in the TyG index <0 exhibited a significantly decreased 13% risk of CKD progression (HR: 0.87, 95% CI: 0.76–0.98) compared to those with a change in the TyG index≥0 group. Subgroup analyses showed that changes in the TyG index significantly increased the risk of CKD progression only in patients with diastolic blood pressure (DBP) <90mmHg. In the path analysis, baseline TyG was associated with follow-up eGFR (the standard regression coefficient was 1.26 [95% CI, 0.45–2.06]).

**Conclusions:**

Our findings suggest that TyG variability may serve as a useful tool for identifying individuals at risk of CKD progression, particularly hypertensive patients with normal DBP levels.

## Introduction

Chronic kidney disease (CKD), with its substantial impact on global health, has become a leading cause of mortality worldwide. Approximately 10–15% of the global adult population is affected by CKD, and the increasing prevalence of CKD is attributable to factors such as population aging, hypertension, and the increasing incidence of diabetes (1–3). Patients with CKD have a higher risk of cardiovascular mortality and end-stage renal disease than do patients without CKD (4). The Renal Disease Burden Study, conducted from 1990 to 2017, revealed 697.5 million cases of CKD worldwide, wherein China and India accounted for nearly one-third of the global burden, with 132 and 115 million cases, respectively (5). Therefore, implementing comprehensive measures at various levels in China is imperative to effectively prevent CKD and manage the occurrence and progression of CKD. Although diabetes-related CKD is the leading cause of CKD in China (6), the prevalence of hypertension-induced CKD should not be underestimated, as China has approximately 245 million adults with hypertension (7). Therefore, identifying biomarkers associated with renal function and monitoring emerging biomarkers are imperative in patients with hypertension.

Insulin resistance (IR) is associated with glomerular hyperfiltration, sodium retention, tubular reabsorption defects, tissue inflammation, and fibrosis (8–10). The association between IR and CKD has garnered considerable attention in recent studies, and IR detection has revealed early metabolic alterations in patients with CKD (11). The triglyceride (TG)–glucose (TyG) index is a logarithmic product of the levels of fasting TG and blood glucose and is commonly used as a substitute index for IR (12) in large-scale population studies. The TyG index, which was initially proposed by Guerrero-Romero et al (13), as a novel alternative marker for IR, is more readily available and cost-effective than the commonly used gold standard (euglycemic–hyperinsulinemic clamp) for IR in clinical practice (14). The TyG index demonstrated a significant association with a positive euglycemic–hyperinsulinemic clamp test, and the validity of the TyG index was comparable to that of the IR index, which has been assessed in the homeostatic model (13). Most previous studies only evaluated the relationship between TyG and CKD at the cross-sectional level (15–17), and cohort studies on CKD progression caused by TyG have been conducted in both patients with diabetes (18, 19) and the general population (20).

Nonetheless, the effect of TyG and its dynamic changes in CKD progression have not been investigated previously. Thus, we posited that baseline TyG serves as a reliable predictor of CKD progression, and that controlling fluctuations in TyG can effectively prevent CKD progression in patients with hypertension. To validate these assumptions, we used data from the China H-type Hypertension Registry Study and monitored the cohort for 52 months.

## Methods

### Study design

Data for this prospective cohort study were acquired from the China H-type Hypertension Registry (registration number: ChiCTR1800017274). The detailed methodology employed in this investigation has been expounded previously (21, 22). Briefly, the China Hypertension Registry Study is an observational study being conducted in Wuyuan, Jiangxi Province, China to reflect real-world conditions and establish a registry cohort to investigate the prevalence, treatment, and related prognostic factors of H-type hypertension. A total of 14,234 patients with hypertension aged ≥18 years were enrolled in this study, after excluding those with psychiatric disorders or short-term residency. In the present study, we conducted a 52-month follow-up of the abovementioned participants. Informed consent was obtained from all enrolled patients. The research protocol was approved by the Ethics Committee of the Second Affiliated Hospital of Nanchang University and the Ethics Committee of the Institute of Biology, Anhui Medical University.

**Figure 1:**
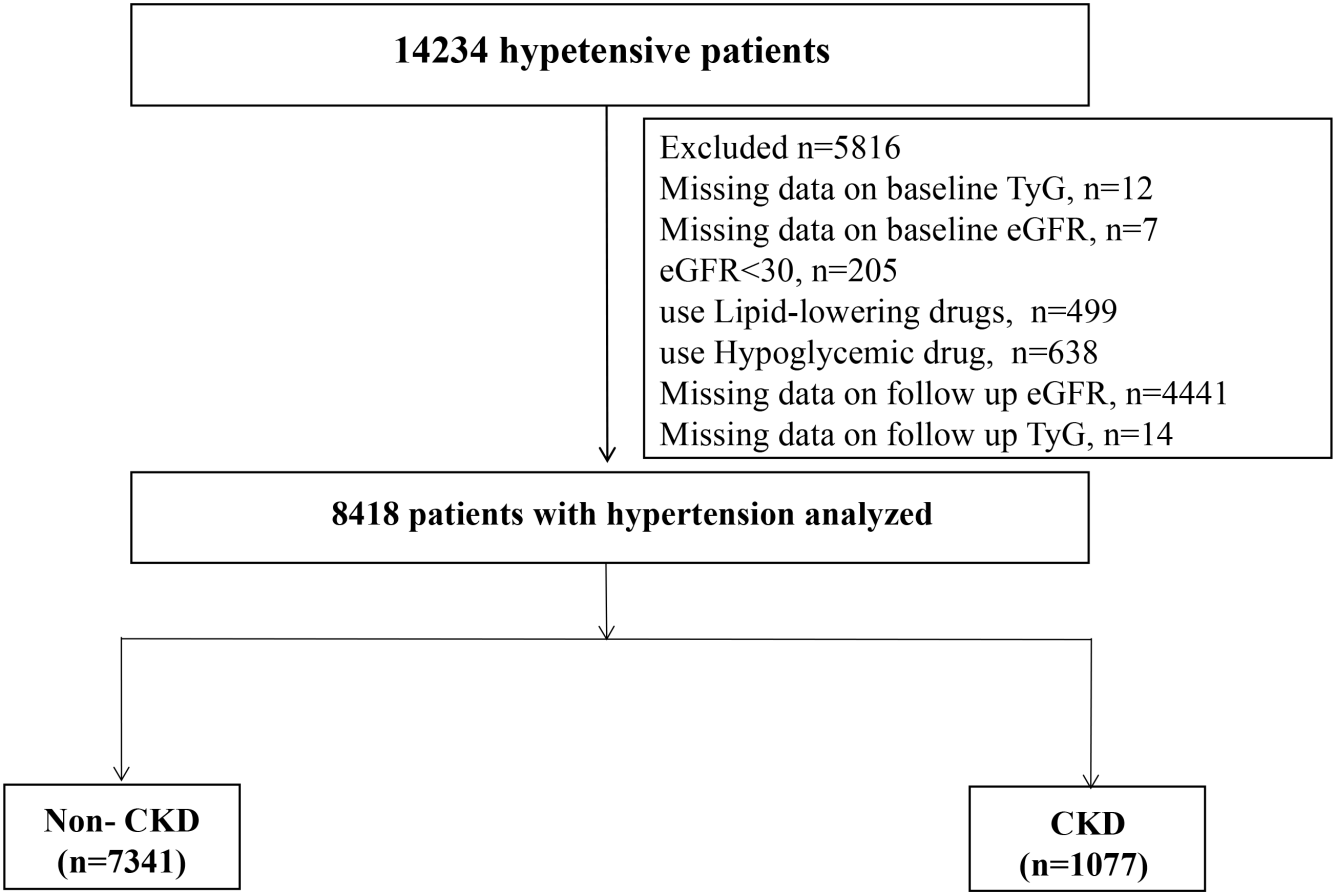
Flow chart of study participants.

Among the 14,234 patients, we excluded 12 individuals with TyG deletion at baseline, 205 patients with estimated glomerular filtration rate (eGFR) <30 mL/min/1.73 m^2^, and 7 patients with eGFR deletion at baseline. We excluded patients who were taking lipid-lowering (n=499) or hypoglycemic (n=638) drugs at baseline. Moreover, 4441 and 14 patients whose eGFR and TyG values, respectively, were missing at the last follow-up were excluded. Finally, 8418 patients were included in this cohort analysis (**Figure 1**).

### Data collection

Baseline data were obtained using face-to-face questionnaires, physical examinations, and laboratory blood tests conducted by trained professionals following a standardized protocol. The questionnaires elicited information on fundamental demographic characteristics, lifestyle factors (history of tobacco and alcohol consumption), medical history (diabetes, coronary heart disease [CHD], and stroke), and medication history (antihypertensive, hypoglycemic, lipid-lowering, and antiplatelet drugs). Physical examinations primarily involved assessing height, weight, and blood pressure (BP). We used an automatic sphygmomanometer to obtain baseline BP readings with the patients seated. Following at least 10 min of rest, two or three measurements were taken at 1-min intervals and averaged. The body mass index (BMI) was computed on the basis of the patients’ weight and height measurements (kg/m^2^).

All blood samples were collected after overnight fasting of 8-12 h and were quickly processed, frozen, and dispatched to the Biaojia Biotechnology Laboratory (Shenzhen, Guangdong Province) for analysis using automated clinical analyzers. TG, total cholesterol (TC), high-density lipoprotein cholesterol (HDL-C), low-density lipoprotein cholesterol (LDL-C), fasting plasma glucose (FPG), homocysteine (Hcy), serum uric acid (SUA), creatinine, and albumin levels were measured in the patients. Serum creatinine was enzymatically determined and calibrated for isotope dilution mass spectrometry traceability with a coefficient of variation of 1.4%. The eGFR was derived using the CKD-EPI equation recommended by the CKD Epidemiology Collaboration (23).

### Outcome and exposure variables

TyG= ln[TG*FBP/2], where the units for TG and FPG are expressed in mg/dL. The exposure variable in this study pertains to the change of TyG index, specifically the difference between the value at last follow-up and baseline TyG. For data analysis purposes, we have set 0 as the cut-off point; a change value of TyG index ≥0 indicates an increase during follow-up, while a change value < 0 denotes a decrease. The study’s outcome variable was CKD progression, which was defined as follows: for patients with an eGFR ≥60 mL/min, a ≥30% reduction in the eGFR, with a final follow-up eGFR <60 mL/min; for patients with an eGFR <60 mL/min, a ≥50% reduction in the eGFR; or terminal renal failure requiring dialysis.

### Statistical analysis

Among the baseline characteristics, the continuous and categorical variables are presented as the mean (standard deviation [SD]) and frequency (proportion), respectively. Differences between patients in the CKD and non-CKD groups were evaluated using a one-way analysis of variance or the chi-square test. A multivariable Cox proportional hazards model was used to estimate the TyG index change and the risk of CKD progression, with adjustments for sex; age; BMI; systolic BP (SBP); diastolic BP (DBP); diabetes; stroke; CHD; antihypertensive and antiplatelet drug use; current smoking; current drinking; and Hcy, TC, LDL-C, SUA, and albumin levels. Variables known as traditional risk factors for CKD and the potential confounders were selected if the effect estimates individually changed by at least 10% (24). Furthermore, restricted cubic splines with four knots were used to flexibly model and visualize the dose-response relationship between changes in TyG and CKD progression. We tested for potential nonlinearity using a likelihood ratio test, while comparing the model with only a linear term against the model with both linear and cubic spline terms (25–28). Subgroup analyses stratified by sex, age, BMI, SBP, DBP, and antihypertensive drug use were performed. Participants (n=8814) with repeated TyG and eGFR assessments constituted a typical cross-lagged panel design, as demonstrated in **Supplementary Figure S1** in the Data Supplement. This design measured the effect size of baseline eGFR on subsequent TyG (β1) and the effect size of baseline TyG on subsequent eGFR (β2) simultaneously, adjusting for the auto-regressive effects.

Statistical significance was defined as a two-sided p-value <0.05 throughout the analysis. We conducted a cross-lagged path analysis using the Mplus software, version 8.4 (Muthén and Muthén, Los Angeles, CA, USA), All other statistical analyses were performed using the statistical package R, version 4.2.3, (http://www.R-project.org, The R Foundation).

## Results

### Participant and baseline characteristics

This prospective cohort study included 8418 patients with hypertension (age, mean ± SD: 63.09 ± 8.65 years), of whom 46.68% were male. During the median follow-up of 48 months, 1077 participants were diagnosed with CKD progression. The baseline study population profiles of the patients with CKD and without CKD are presented in **Table 1**. Patients with CKD tended to be older males and current smokers but not current drinkers. Patients with CKD exhibited higher BMI and SBP values, higher levels of Hcy and SUA, and a higher incidence of diabetes, CHD, and antihypertensive drug use than did patients without CKD. Conversely, patients without CKD had a lower DBP value and albumin level than did those with CKD. However, no significant intergroup differences were observed in the FPG, TC, TG, HDL-C, and LDL-C levels; stroke prevalence; and use of antiplatelet drugs.

**Table 1:** Baseline characteristics of study participants^1^.

Variable	Total	Non- CKD	CKD	*P* value
N	8418	7341	1077	
Age,y	63.08 ± 8.65	62.35 ± 8.57	68.07 ± 7.48	<0.001
Male, n(%)	3932 (46.71%)	3374 (45.96%)	558 (51.81%)	<0.001
Current smoker, n(%)	2210 (26.26%)	1898 (25.86%)	312 (28.97%)	0.030
Current drinker, n(%)	1912 (22.72%)	1698 (23.14%)	214 (19.87%)	0.017
BMI, kg/m^2^	23.71 ± 3.49	23.76 ± 3.49	23.39 ± 3.48	0.001
SBP, mmHg	148.50 ± 17.25	148.20 ± 17.00	150.55 ± 18.77	<0.001
DBP, mmHg	89.43 ± 10.40	89.69 ± 10.26	87.69 ± 11.14	<0.001
Hcy,μmol/L	17.36 ± 10.08	16.77 ± 9.52	21.38 ± 12.58	<0.001
FPG, mmol/L	6.04 ± 1.33	6.02 ± 1.29	6.16 ± 1.60	0.002
TC, mmol/L	5.20 ± 1.09	5.20 ± 1.08	5.17 ± 1.16	0.282
TG, mmol/L	1.80 ± 1.23	1.80 ± 1.22	1.83 ± 1.29	0.386
HDL-C, mmol/L	1.58 ± 0.43	1.58 ± 0.43	1.57 ± 0.44	0.418
LDL-C, mmol/L	3.01 ± 0.80	3.02 ± 0.79	2.98 ± 0.85	0.123
SUA, mmol/L	415.93 ± 118.52	406.38 ± 112.89	481.07 ± 134.43	<0.001
ALB, mmol/L	46.80 ± 3.98	46.94 ± 3.95	45.88 ± 4.11	<0.001
diabetes, n (%)	1154 (13.71%)	977 (13.31%)	177 (16.43%)	0.005
stroke, n (%)	446 (5.30%)	377 (5.14%)	69 (6.41%)	0.082
CHD, n (%)	365 (4.34%)	300 (4.09%)	65 (6.04%)	0.003
Antihypertensive drugs, n (%)	5380 (63.93%)	4603 (62.72%)	777 (72.14%)	<0.001
Antiplatelet drugs, n (%)	140 (1.66%)	116 (1.58%)	24 (2.23%)	0.120

^1^Values are mean ± SD, or n (%) for categorical variables.

BMI, body mass index; SBP, systolic blood pressure; DBP, diastolic blood pressure; Hcy, homocysteine; HDL-c, high-density lipoprotein cholesterol; LDL-C, low-density lipoprotein cholesterol.

### TyG index change and CKD progression

**Figure 2:**
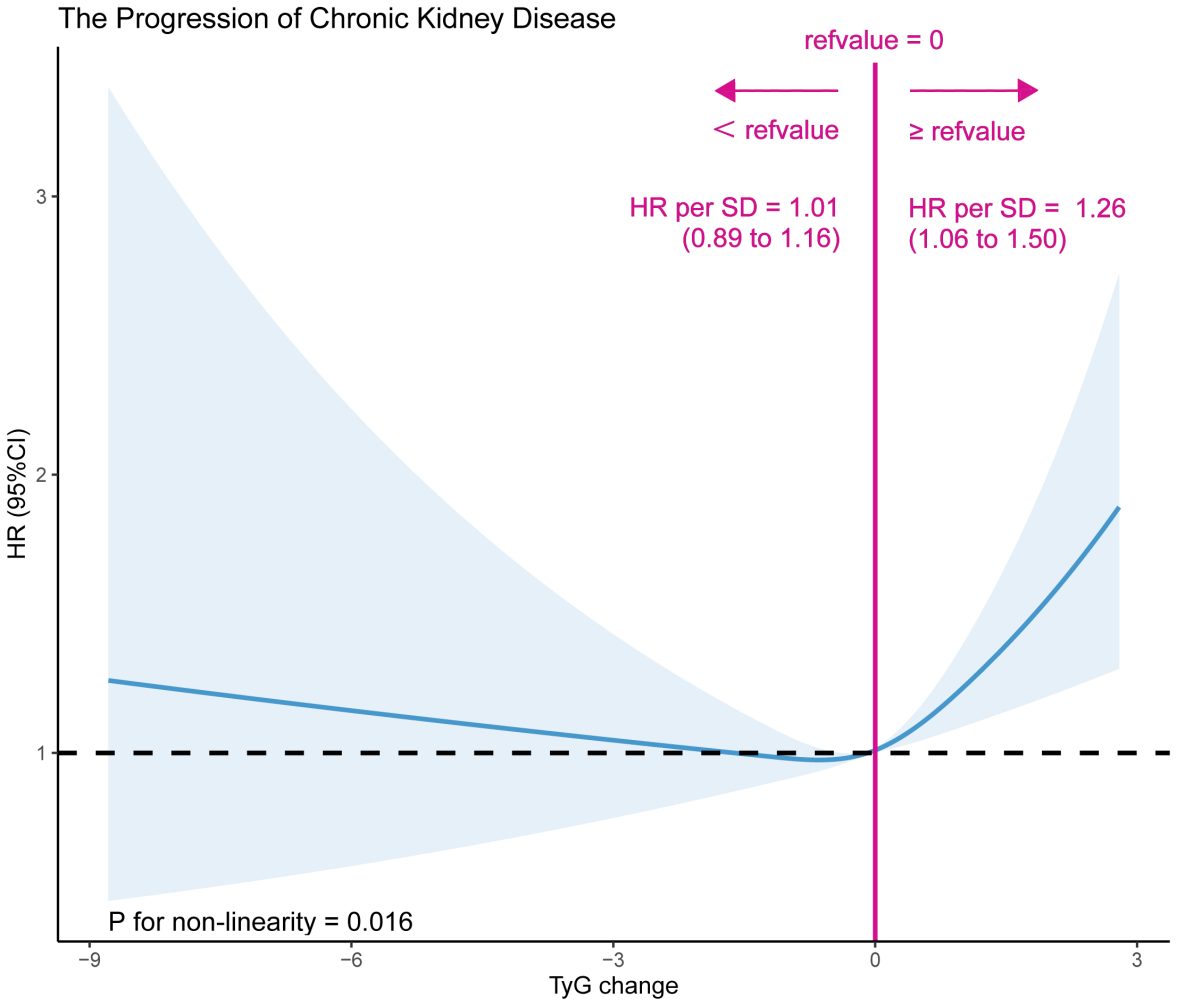
A dose-effect response fitting curve association between TyG index change and CKD progression. A nonlinear association between TyG index change and CKD progression was found. The solid line and dashed line represent the estimated values and their corresponding 95% confidence interval. Adjustment factors included sex, age, BMI, SBP, DBP, diabetes, stroke, CHD, antihypertensive, antiplatelet drugs, current smoke,current drink, Hcy, TC, LDL-C, SUA, albumin.

A dose-effect response fitting curve was used with restricted cubic splines, as shown in **Figure 2**, to demonstrate the correlation between changes in the TyG index and CKD progression. The risk of CKD progression was relatively flat until an approximately 0 value for changes in the TyG index, following which it began to increase rapidly (P for non-linearity = 0.016). With >0 increase in changes in the TyG index, the HR per SD was 1.26 (1.06–1.50). Moreover, the Cox proportional hazards model was used to ascertain the association between changes in the TyG index and the risk of progressive CKD (**Table 2**). In the stepwise adjusted model, we observed a significant association between the change in the TyG index and CKD progression. The HR (95% confidence interval [CI]) for each additional unit of change in the TyG index was 1.17 (1.09–1.26), 1.15 (1.07–1.24), and 1.11 (1.03–1.20) in Models 1, 2, and 3, respectively. Using 0 as the tangent point, we divided the changes in the TyG index into two cohorts and examined the impact of decreased TyG index on CKD progression. In the fully adjusted Model 3, patients with a change in the TyG index<0, compared with those with a change in the TyG index≥0, exhibited a significantly reduced 13% risk of CKD progression (HR [95% CI]: 0.87 [0.76–0.98]). Additional Cross-Lagged Analysis for TyG and eGFR was based on 8418 participants, Results that showed baseline TyG was associated with follow-up eGFR (the standard regression coefficient was 1.26 [95% CI, 0.45–2.06]). In contrast, the standard regression coefficient of baseline TyG for follow-up eGFR (β=-2.06 [95% CI, −4.13 to 0.01]) was not significant (**Supplementary Figure S1**). This indicated that increase in TyG preceded increase in eGFR.

**Table 2:** Hazard ratio of CKD according to continuous or tertiles of surrogate markers of IR.

Variables	N	Events (%)	CKD *HR* (95%CI), P-value		
			Model 1	Model 2	Model 3
Per 1 unit TyG change	8432	1077 (12.79%)	1.17 (1.09, 1.26) <0.0001	1.15 (1.07, 1.24) 0.0002	1.11 (1.03, 1.20) 0.004
Categorical variable					
change in TyG ≥0	3874	500 (12.91%)	1 (reference)	1 (reference)	1 (reference)
change in TyG <0	4544	577 (12.70%)	0.80 (0.71, 0.91) 0.0004	0.82 (0.73, 0.93) 0.002	0.87 (0.76, 0.98) 0.024

Model 1 was adjusted for age, sex.

Model 2 was adjusted for sex, age, BMI, SBP, DBP.

Model 3 was adjusted for sex, age, BMI, SBP, DBP, diabetes, stroke, CHD, antihypertensive, antiplatelet drugs, current smoke,current drink, Hcy, TC, LDL-C, SUA, albumin.

### Subgroup analysis

**Figure 3:**
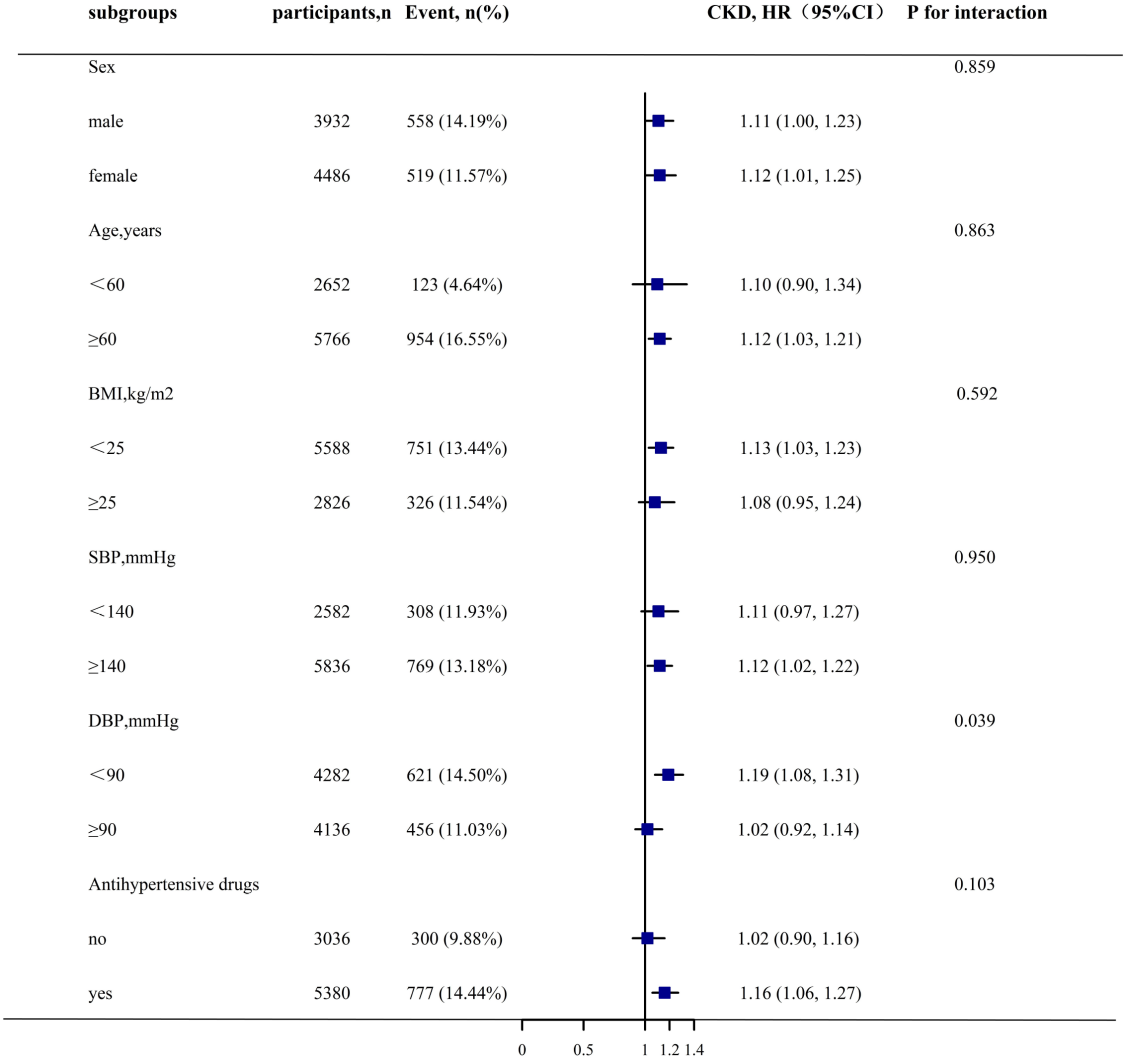
Stratified Analyses by Potential Modifiers of the Association between TyG index change and CKD progression*. *Each subgroup analysis adjusted for sex, age, BMI, SBP, DBP, diabetes, stroke, CHD, antihypertensive, antiplatelet drugs, current smoke,current drink, Hcy, TC, LDL-C, SUA, albumin except for the stratifying variable.

Stratification and interaction tests were employed to evaluate whether the association between changes in the TyG index and CKD progression was potentially modifiable in the subgroups. Changes in the TyG index significantly increased the risk of CKD progression only in the patients with DBP <90 mmHg, compared with that in the patients with DBP ≥90 mmHg (HR [95% CI]: 1.19 [1.08–1.31] vs. 1.02 [0.92–1.14]; P for interaction = 0.039). However, the relationship between the changes in the TyG index and CKD progression remained consistently positive across the subgroups stratified by sex (male vs. female), age (<60 vs. ≥60 years), BMI (<25 vs. ≥25 kg/m^2^), SBP (<140 vs. ≥140 mmHg), and antihypertensive medication use (no vs. yes) (P for interaction >0.05) (**Figure 3**).

## Discussion

This cohort study is the first to investigate the association between changes in the TyG index and CKD progression in patients with hypertension. The findings indicate that TyG variability is an independent predictor of CKD progression, with more pronounced adverse effects observed in patients with normal DBP.

The TyG index was initially employed to assess its association with cardiac metabolic disorders. Specifically, Liang et al. conducted a meta-analysis of 41 cross-sectional and longitudinal cohort studies to investigate the impact of the TyG index on coronary heart disease (CHD). Their findings revealed a positive correlation between increasing TyG index levels and an elevated relative risk of CHD (29). The systematic review conducted by Khalaji et al. encompassing 30 cohort studies with a total of 772,809 participants also demonstrates that an elevated TyG index is associated with an increased risk of heart failure in individuals diagnosed with CHD, diabetes, and pre-existing heart failure (30). These two meta-analyses conducted by Wang (31) and Ling (32) provide further support for the TyG index as an independent risk factor for nonalcoholic fatty liver disease. Additionally, a meta-analysis of 13 cohort studies demonstrated that the TyG index is a significant predictor of diabetes risk (33). The meta-analysis conducted by Xiao et al. revealed that an elevated TyG index was associated with an increased prevalence of CKD, independent of the established risk factors (34). In recent years, the utility of the TyG index to evaluate the risk of IR in adults has been demonstrated (35). Several previous investigations have examined the impact of the TyG index on CKD; however, they predominantly had a cross-sectional design. Liu et al. (15) found that elevated TyG levels were associated with a 91% (95% CI 1.29–2.85) increase in the prevalence of diabetic nephropathy among patients with type 2 diabetes, with statistical significance. However, a cross-sectional study conducted by Ou et al. (17) on 1872 outpatients with diabetes in the Taiwan Province found no significant correlation between the TyG index and renal failure (eGFR <30 mL/min/1.73 m2). Lv et al. (16)investigated the association between the TyG index and both pre-existing CKD and incident CKD in patients with type 2 diabetes, reporting that baseline TyG levels are a promising predictor of diabetic nephropathy. Similarly, our group’s previous cross-sectional analysis demonstrated a significant association between elevated TyG levels and the presence of CKD (odds ratio: 1.42, 95% CI 1.24–1.64) in patients with hypertension (36). Owing to the inherent limitations of the cross-sectional study design, a causal relationship between the TyG index and CKD could not be inferred. However, there is a paucity of longitudinal studies examining the association between the TyG index and CKD progression. Using electronic medical records, Duan et al. (19) retrospectively gathered data from 179 in patients with type 2 diabetes mellitus and CKD and demonstrated that a high baseline TyG level significantly increased the risk of CKD progression by 79.4% (HR [95% CI] 1.794 [1.026–3.137]; P = 0.040). Furthermore, Low (18) discovered a robust correlation between the baseline TyG index and CKD progression in 1571 patients with type 2 diabetes after an extended follow-up period of 8.6 years.

Previous studies have frequently confirmed the association between TyG and CKD or CKD progression in patients with diabetes, and this may be attributed to IR - a distinctive marker of diabetes that better reflects metabolic disorders within the body. However, patients with hypertension experience not only diabetes but also metabolic disorders due to the presence of high BP as a component of metabolic syndrome (37, 38). We observed a similar phenomenon in the patients with hypertension, broadening the research population subject to the impact of the TyG index on CKD; this has substantial implications for comprehensive CKD patient management. The primary discoveries of this investigation are as follows. For the first time, a dynamic TyG index has been used to predict the progression of CKD in patients with hypertension. When the TyG change was considered a continuous variable, we observed that an increase in the TyG index change was an independent and significantly elevated risk factor for CKD progression, regardless of the presence of CKD. Moreover, we set the change of TyG to 0, where a positive value denotes an increase in the TyG index and a negative value indicates a decrease from the baseline. Specifically, a decrease in both baseline and follow-up TyG index values conferred renal protection against CKD progression. This finding could play an influential role in clinical practice by enabling dynamic, real-time monitoring of the TyG index and an accurate evaluation of the risk of CKD in patients with hypertension. Furthermore, the subgroup analysis revealed a correlation between DBP-adjustable TyG index changes and CKD progression. Specifically, our findings indicate that among patients with DBP <90 mmHg, an increase in the TyG index was associated with a significantly higher risk of CKD progression. However, this phenomenon was not observed in patients with DBP ≥90 mmHg, possibly owing to the masking effect of increased DBP on the association between the TyG index change and CKD. Therefore, a positive correlation can easily be detected in patients with normal DBP. This implies that, in our clinical practice, the treatment priorities may vary for different patients with DBP.

The pathophysiological mechanisms underlying the association between the TyG index and CKD are modulated by IR, which affects the metabolic processes in the body. Insulin signaling is essential for podocyte function and the maintenance of glomerular integrity (39); within the kidney, multiple insulin-sensitive cell types express insulin receptors, and specific knockdown of these receptors in podocytes or proximal tubules results in proteinuria, renal pathology, and hyperglycemia (40, 41). Shimobayashi et al. (42)demonstrated that IR triggers adipose tissue inflammation by inhibiting insulin signaling pathways and increasing the production of monocyte chemoattractant protein 1. The inflammatory activation of M2 macrophages in the adipose tissue induces the release of proinflammatory cytokines, which cause glomerular endothelial dysfunction and ultimately lead to CKD (43, 44). The verified association between IR and the significant increase in CKD has established a mechanistic link. In our study, we utilize a dynamic TyG index as an exposure factor to reflect changes in the body’s IR. Therefore, an elevated TyG index signifies increased IR, which can contribute to the progression of CKD and establish a positive feedback loop with worsening degrees of IR.

### Strengths and limitations

The findings of this study are noteworthy, as they stem from a prospective cohort study that was conducted on a sizable population of participants with hypertension in China. The extended follow-up period enabled us to longitudinally track the eGFR of the participants, particularly in those with a relatively well-preserved eGFR at baseline. This underscores the importance of managing CKD progression through the dynamic monitoring of TyG index changes and stratification of CKD risk across diverse DBP populations to optimize the clinical outcomes. This study also had a few limitations. Our study was conducted solely on patients with hypertension from rural regions in China and, therefore, cannot be generalized to community-dwelling patients with hypertension residing in urban areas. Additionally, the study’s focus on hypertensive patients may limit its generalizability to the broader population with CKD. Although the possibility of unmeasured confounding variables cannot be entirely dismissed, the magnitude of the observed effect makes it improbable that such variables completely accounted for the association that was identified.

## Conclusions

Our findings suggest that TyG index variability may serve as a useful tool for identifying individuals who are at risk of CKD progression, particularly among patients with hypertension with normal DBP levels. The TyG index, a simple and cost-effective biomarker, has substantial implications in clinical practice and public health policy for preventing CKD progression and its associated complications.

## Data availability statement

The data that support the findings of this study are available from the corresponding author upon reasonable request. Requests to access these datasets should be directed to XC, xiaoshumenfan126@163.com.

## Ethics statement

The studies involving humans were approved by The research protocol was approved by the Ethics Committee of the Second Affiliated Hospital of Nanchang University and the Ethics Committee of the Institute of Biology, Anhui Medical University. The studies were conducted in accordance with the local legislation and institutional requirements. The participants provided their written informed consent to participate in this study.

## Author contributions

CY: Data curation, Writing – original draft, Writing – review & editing. YS: Investigation, Software, Writing – original draft, Writing – review & editing. TW: Data curation, Writing – review & editing. LZ: Data curation, Investigation, Writing – review & editing. WZ: Data curation, Investigation, Writing – review & editing. HB: Funding acquisition, Methodology, Supervision, Writing – review & editing. XC: Funding acquisition, Methodology, Supervision, Writing – review & editing.

## Supplementary material

Supplementary Figure 1 Cross-lagged relationships between TyG and eGFR (N = 8418) Note. Values represented standardized path coefficients. Curved lines reflect correlation between exogenous factors or disturbances of endogenous factors. Numbers in round brackets represent variances of the residual error. The circles represent the residual terms.
