# Supplementary material for: Triglyceride–glucose index change and chronic kidney disease progression in a Chinese hypertensive population
Source: Front Endocrinol (Lausanne). 2024 Feb 9;15:1342408. doi: 10.3389/fendo.2024.1342408 (PMC10893760; doi:10.3389/fendo.2024.1342408)
Supplement: Supplementary Figure 1 — Cross-lagged relationships between TyG and eGFR (N = 8418) Note. Values represented standardized path coefficients. Curved lines reflect correlation between exogenous factors or disturbances of endogenous factors. Numbers in round brackets represent variances of the residual error. The circles represent the residual terms. [file Image_1.pdf]

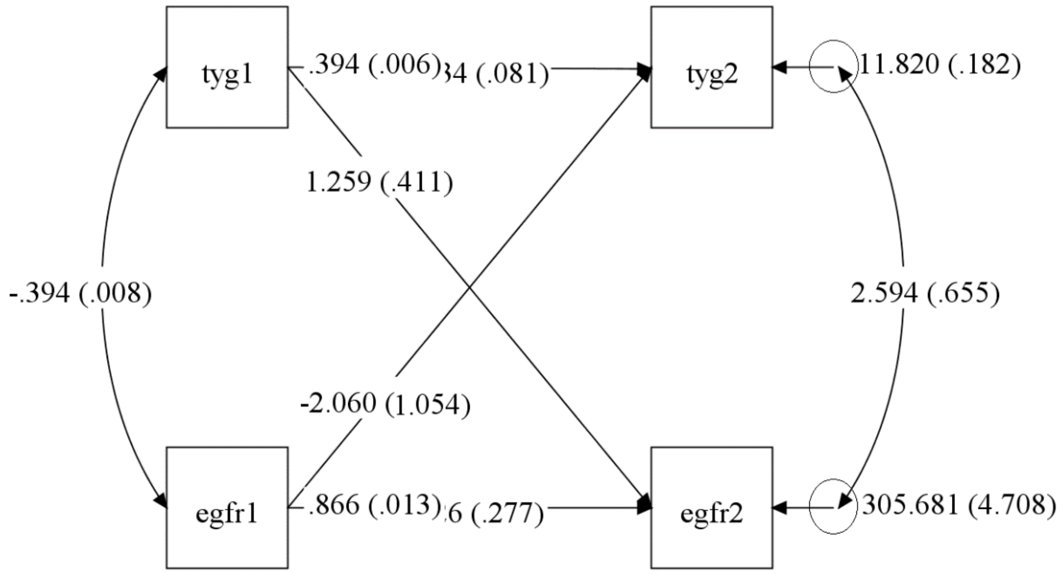

Figure S1 Cross-lagged relationships between TyG and eGFR (N = 8418) Note. Values represented standardized path coefficients. Curved lines reflect correlation between exogenous factors or disturbances of endogenous factors. Numbers in round brackets represent variances of the residual error. The circles represent the residual terms.
